# Inhibitory Effects of Phylligenin and Quebrachitol Isolated from *Mitrephora vulpina* on Platelet Activating Factor Receptor Binding and Platelet Aggregation

**DOI:** 10.3390/molecules15117840

**Published:** 2010-11-03

**Authors:** Bushra Abdulkarim Moharam, Ibrahim Jantan, Juriyati Jalil, Khozirah Shaari

**Affiliations:** 1 Faculty of Pharmacy, Universiti Kebangsaan Malaysia, Jalan Raja Muda Abdul Aziz, Kuala Lumpur 50300, Malaysia; 2 Institute of Bioscience, Universiti Putra Malaysia 43400 Serdang, Selangor, Malaysia

**Keywords:** *Mitrephora vulpina*, phylligenin, quebrachitol, platelet activating factor (PAF) antagonist, antiplatelet activity

## Abstract

Phylligenine, together with quebrachitol, stigmasterol and two aporphine alkaloids—oxoputerine and liriodenine—were isolated from the twigs of *Mitrephora vulpina* C.E.C. Fisch. They were evaluated for their ability to inhibit platelet activating factor (PAF) receptor binding to rabbit platelets using ^3^H-PAF as a ligand and their antiplatelet aggregation effect in human whole blood induced by arachidonic acid (AA), collagen and adenosine diphosphate (ADP). Of all the compounds tested, phylligenin and quebrachitol exhibited potent and concentration-dependent inhibitory effects on PAF receptor binding, with IC_50_ values of 13.1 and 42.2 µM, respectively. The IC_50_ value of phylligenin was comparable to that of cedrol (10.2 µM), a potent PAF antagonist. Phylligenin also showed strong dose-dependent inhibitory activity on platelet aggregation induced by AA and ADP.

## 1. Introduction

Platelet activating factor (PAF) is a potent glycerophospholipid mediator which is involved in a number of dose-dependent physiological functions such as platelet and neutrophil aggregation, inducement of changes in vascular permeability, anaphylaxis and hypotension [1]. PAF has also been reported to be involved in many pathological conditions such as bronchoconstriction-induced asthma, hypertacute organ-transplant rejection, inflammation, allergic reaction, thrombosis, endotoxin shock, cardiac anaphylaxis and gastrointestinal ulceration [2]. The activation of the PAF-mediated pathogenic conditions has been reported to be dependent on the specific binding between PAF and receptors found in a variety of cell membranes including those from platelets, polymorphonuclear leukocytes and membranes from several types of tissues [3]. Thus specific antagonists of PAF, *i.e.* agents which inhibit the specific binding to the receptor have been extensively sought to be used as leads in the development of therapeutic agents in a variety of inflammatory, respiratory, immunological and cardiovascular disorders. 

Platelets interact with activated plasma clotting factors at the site of blood vessel injury in the normal haemostatic process. Platelet aggregation plays an important role in the formation of rapidly progressing atherosclerotic lesions and acute arterial thrombosis [4]. Platelet aggregation is induced by the action of endogenous agonists such as arachidonic acid (AA), adenosine diphosphate (ADP), PAF, collagen and thrombin [5]. Antiplatelet agents can inhibit platelet activation by many mechanisms and useful in the treatment of platelet hyperactivity especially to reduce the risk of serious ischaemic events in several cardiovascular disease states including stroke, myocardial infarction, unstable angina and following coronary artery bypass surgery [6]. Aspirin may cause gastric erosions and gastric ulcers and patients may present with anaemia and gastric haemorrhage. Other current antiplatelet drugs such as clopidogrel and ticlopidine still have considerable limitation in their mode of action and efficacy [7]. Thus, the search for more potent and safer platelet inhibitors has continued.

*Mitrephora* (Family: Annonaceae) is a genus of about 48 species of shrubs and small to large trees. The diversity of this plant is centered in Borneo and the Philippines but also distributed throughout China in the north, India in the west, and Australia (Queensland) in the south-east [8]. A few of the *Mitrephora* species have been investigated for their chemical components, including *M. celebica, M. maingayi, M. thorelii, M. tomentosa, M. diversifolia, M. zippeliana* and *M. glabra* [9,10,11,12,13,14,15,16,17,18]. As in other Annonaceae species, alkaloids, diterpenoids, polyacetylenic acids and esters have been reported from various *Mitrephora* species. *M. celebica*, *M. diversifolia*, *M. glabra* and *M. thorelii* have been investigated for various biological activities which include antimicrobial, anticancer and antimalarial properties [14,15,16,17,18]. However, to our knowledge there is no report on the chemical constituents or biological activity of *M. vulpina.*

In our screening study to identify new natural PAF antagonists and antiplatelet agents from tropical plants, we observed that the methanol extract of the twigs of *Mitrephora vulpina* C.E.C. Fisch showed strong inhibitory effects on PAF receptor binding to rabbit platelets using ^3^H-PAF as a ligand (52% inhibition) and antiplatelet aggregation effect in human whole blood induced by arachidonic acid (AA), collagen and adenosine diphosphate (ADP) (> 60.0% inhibition). In this paper, we report on the isolation of phylligenin, together with quebrachitol, stigmasterol and the two aporphine alkaloids oxoputerine and liriodenine from this plant and their ability to displace ^3^H-PAF-specific binding from washed rabbit platelets and inhibit platelet aggregation in human whole blood.

## 2. Results and Discussion

### 2.1. Isolation and identification of compounds

**Figure 1:**
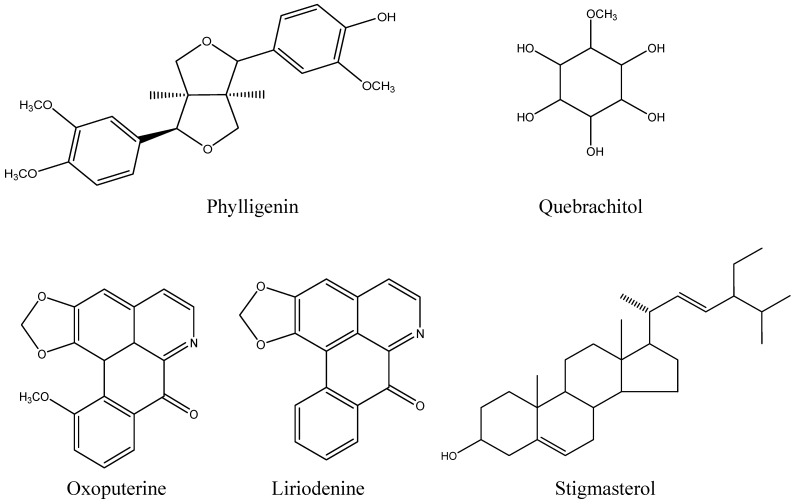
Structures of compounds from *Mitrephora vulpina.*

In this study, five known compounds have been isolated from the twigs of *Mitrephora vulpina* by various chromatographic techniques. The compounds were identified as oxoputerine [19], liriodenine [20], stigmasterol [21], quebrachitol [22] and phylligenin [23] by comparison of their physicochemical and spectroscopic properties with literature values. The bis-tetrahydrofuran type lignan, phylligenin, was recorded for the first time from the genus *Mitrephora* (see Figure 1 for the structures of the compounds).

### 2.2. Inhibition of PAF receptor binding

**Figure 2:**
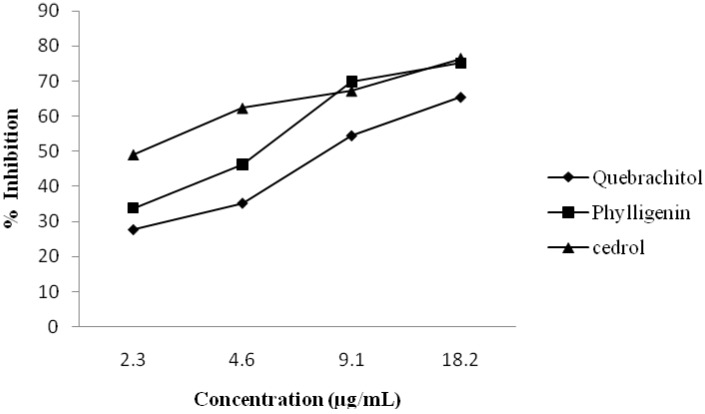
Inhibitory effect of phylligenine, quebrachitol and cedrol on ^3^H-PAF binding to receptor on rabbit platelets at various concentrations. Each point represents the mean of three experiments, each in triplicate. Standard deviation of the mean are indicated as vertical bars.

The methanol extract of the twigs of *Mitrephora vulpina* showed strong inhibitory effect at 18.2 µg/mL on PAF receptor binding to rabbit platelets using ^3^H-PAF as a ligand (52% inhibition). Table 1 shows that phylligenin and quebrachitol, a cyclitol, exhibited a significant inhibitory effect on PAF receptor binding with 75.2 and 65.5% inhibition respectively, at 18.2 µg/mL. The inhibitory effects of the compounds were evaluated at various concentrations and the IC_50_ values of phylligenin and quebrachitol were determined by probit analysis as 13.1 and 42.2 µM, respectively. Figure 2 shows the dose-dependent responses of the compounds and cedrol, a potent PAF antagonist used in this study as a positive control [24]. The IC_50_ value of phylligenin was comparable to that of cedrol (10.6 µM). The result was in agreement with the results of previous study that showed plant species rich in bis-tetrahydrofuran type lignans have high antagonistic effect against PAF receptor binding specially those containing at least one 3,4-dimethoxyphenyl group [25]. Recently, phylligenin has been reported to exhibit anti-inflammatory effect as it inhibited cyclooxygenase-2 (COX-2)-mediated prostaglandin E_2_ and iNOS mediated nitric oxide synthesis from lipopolysaccharide-treated RAW 264.7 cell [23]. The PAF antagonistic activity of quebrachitol (2-O-methyl-L-chiro-inositol), a sugar like natural compound, is also reported for the first time here. The compound has been reported to have peroxynitrite scavenging, cytoprotective and laxative effects [26,27,28].

**Table 1:** Inhibitory effect of the compounds isolated from *Mitrephora vulpina* on PAF receptor binding to rabbit platelets at 18.2 µg/mL and their IC_50_ values.

Sample	% inhibition	IC_50_ (µM)
Oxoputerine	34.6 ± 6.5	
Liriodenine	27.7 ±2.7	
Stigmasterol	25.7 ± 4.1	
Phylligenine	75.2 ± 7.3*	13.1 ± 2.5 (4.8 ± 0.4)
Quebrachitol	65.5 ± 5.0*	42.2 ± 1.6 (8.2 ± 1.1)
Cedrol	78.0 ± 1.8	10.6 ± 1.2 (2.5 ± 0.2)

Values are calculated from at least three experiments and presented as means ± SD. *P < 0.05 as compared with cedrol. The IC_50_ values in µg/mL are presented in parentheses.

### 2.3. Inhibition of platelet aggregation

The methanol extract of the twigs of *Mitrephora vulpina* showed strong antiplatelet aggregation activity at 100 µg/mL in human whole blood *in vitro* exhibiting > 60.0% inhibition against AA-, collagen and ADP-induced aggregation. Table 2 shows the antiplatelet aggregation activity of the compounds in human whole blood. Among the compounds tested, only phylligenin showed significant antiplatelet activity on AA- and ADP-induced aggregation. Phylligenin showed a dose-dependent response, *i.e*. as the concentration of the compound increased, the % inhibition increased. The IC_50 _values of phylligenin against AA- and ADP-induced aggregation were 230.6 and 121.8 μM, respectively. The IC_50_ value of phylligenin against AA-induced aggregation was higher than that of acetyl salicylic acid (24.8 μM), a positive control in the bioassay [29]. 

**Table 2:** Percentage inhibition of isolated compound of *Mitrphora vulpina* and aspirin on platelet aggregation of human whole blood induced by AA (0.5 mM), collagen (2 μg/mL) and ADP (10 μM) and their IC_50_ values.

Sample	µg/mL	Inhibition %		
		AA	ADP	Collagen
Oxoputerine	100	6.57 ± 2.0	11.3 ± 1.1	25.2 ± 3.8
Liriodenine	100	38.9 ± 7.1	30.1 ± 7.1*	29.4 ± 2.5
Stigmasterol	100	25.7 ± 0.3	24.8 ± 0.2	8.2 ± 3.3
Phylligenin	100	51.2 ± 1.7*	66.1 ± 7.0*	22.1 ± 3.8
	50	43.6 ± 4.6	50.8 ± 1.7	
	25	29.5 ± 6.6	39.9 ± 6.1	
	12.5	20.7 ± 0.3 (230.6 ± 0.4)	24.7 ± 1.5 (121.8 ± 5.7)	
Quebrachitol	100	10.3 ± 2.0	3.3 ± 0.9	11.8 ± 4.1
Acetylsalicylic acid	25	99.7 ± 0.3*	48.8 ± 8.7*	35.2 ± 4.3*
	12.5	79.4 ± 2.8		
	6.25	62.4 ± 5.3		
	3.13	51.8 ± 3.8		
	1.5	30.7 ± 0.0(24.8 ± 4.5)		

Values are presented as means ± SD (n = 3). *P < 0.05 as compared with control. IC_50_ values in µM are presented in parentheses.

## 3. Experimental

### 3.1. General

Melting points were determined using an Electrothermal melting point apparatus model 9100. Ultraviolet (UV) spectra were obtained on Shimadzu UV-1601 spectrophotometer. IR spectra were recorded on a Perkin-Elmer GX spectrophotometer. Electron impact Mass spectra (EIMS) were performed on Micromass VG 7035 mass spectrometer at 70 ev. The ^1^H-NMR (400MHz) and ^13^C-NMR (100 MHz) spectra were recorded on a JOEL NMR spectrometer in CDCl_3_ or CD_3_OD with TMS as internal standard. Copies of the original spectra are available for reference.

### 3.2. Plant materials

The twigs of *Mitrephora vulpina* were collected in Universiti Kebangsaan Malaysia Forest Reserve in June 2005 and identified by Dr Latif Mohammad, Faculty of Science and Technology, Universiti Kebangsaan Malaysia (UKM). A voucher spacimen (No. AZ 6889) was deposited at the Herbarium of the Faculty of Science and Technology, UKM, for further reference.

### 3.3. Extraction and isolation of compounds

Dried ground twigs of *M. vulpina* (462.2 g) was extracted successively with hexane (3 × 2 L), ethyl acetate (3 × 2 L) and methanol (3 × 2 L) using a Soxhlet apparatus. The extracts were then concentrated using a rotatory evaporator to yield hexane (5.1 g, 1.1 %), ethyl acetate (8.12 g, 1.8 %) and methanol (23.9 g, 5.2%) extracts, respectively. The extracts were subjected to silica gel vacuum liquid chromatography (VLC), column chromatography (CC) and preparative TLC to afford five known compounds. The hexane extracts yielded stigmasterol (an amorphous powder, 20 mg), the ethyl acetate extract afforded phylligenin (a pale green crystals, 303.7 mg) while quebrachitol (a crystalline white powder, 45 mg), liriodenine (a yellow crystal, 20 mg) and oxoputerine (an orange powder, 16 mg) were isolated from the methanol extract. The compounds were identified by comparison of their physicochemical and spectroscopic properties with literature values. 

### 3.4. PAF receptor binding inhibitor assay

Radiolabelled PAF (1-O-[^3^H]-octadecyl-2-acetyl-sn-glycero-3-phosphocholine soluble in ethanol/toluene (1:1) and with a specific activity of 60 Ci/mmol was purchased from American Radiolabeled Chemicals (St. Louis, MO, USA). Unlabeled PAF and cedrol were obtained from Sigma Chemical Co. (St. Louis, MO, USA). The use of rabbit blood was approved by the Animal Ethical Committee of UKM (approval no. FSKB/2007/Juriyati/10-July/192). The assay was carried out according to the modified method of Valone *et al*. [30]. The reaction mixture consisted of 200 μL of washed rabbit platelet suspension, 25 μL of ^3^H-PAF (2.0 nM) with or without 25 μL unlabelled PAF (2.0 µM) and 25 μL of the compound (200 µg/mL) or control solution. The final concentrations of the compounds in the reaction mixtures were 18.2, 9.1, 4.5, 2.3 μg/mL. The final concentration of DMSO (control) in the reaction mixture was fixed at 0.1% to avoid interference with the receptor binding studies. The reaction mixture with 0.1% DMSO in saline was used as a control and cedrol was used as a positive control. The reaction mixture was incubated at room temperature for 1 h. The free and bound ligands were separated by filtration technique using Whatman GF/C glass fibre filters. The radioactivity was measured by scintillation counter (Packard Tri-Carb, models 2100TR/2300TR, Germany). The difference between the total amounts of ^3^H-PAF bound in the absence and in the presence of excess unlabelled PAF was defined as specific binding of ^3^H-PAF. The IC_50_ values of the samples were obtained from at least three independent determinations. 

### 3.5. Antiplatelet aggregation assay

Collagen, ADP and AA were products of Chrono-Log Corp. (Haverton, PA, USA). The antiplatelet activity was performed as described by Jantan *et al*. [31]. The use of human blood was approved by the Ethics Committee of Universiti Kebangsaan Malaysia (UKM; approval no. FF-120-2007). Briefly, blood was taken by venipuncture from healthy human volunteers based on the criteria that that they were nonsmokers and had not taken any medications within the last two weeks, including aspirin, and had not taken any food within the last 8 h. The number of platelets in 1 mm^3^ of blood was determined by a platelet counter (Model PLT-4, Chrono-Log Corp., Havertown, PA) and blood with the normal range between 2 × 10^5^ and 3 × 10^5^ platelets was used. Five µL of each compound (20 µg/µL, in DMSO) was added only after 2 min of the incubation. Then, the inducers, collagen (2 µg/mL), ADP (10 µM) or AA (0.5 mM), was added to initiate aggregation. The final volume of the reaction mixture was one mL. The final concentrations of the compound in the mixture were 100, 50, 25 and 12.5 μg/mL. The platelet aggregation was measured by a Whole Blood Lumi-Aggregometer (Chrono-Log Corp., Havertown, PA) using an electrical impedance method. The mean platelet aggregation in whole blood was measured as a change in impedance over 6 min after the addition of the inducers by comparison to that of a control group impedance. A mixture containing 0.5% DMSO in the diluted whole blood was used as control. Acetylsalicylic acid, a potent cyclooxygenase inhibitor, was used as a positive control in the bioassay. The final concentration of DMSO in the whole blood was 0.5% to eliminate the effect of the solvent on the aggregation. 

### 3.6. Statistical analysis

All the data were analysed using *Statistically Package for Social Sciences* (SPSS) version 15.0. Each sample was measured in triplicate and the data are presented as means ± standard deviation (SD). Probit programme was used to determine the IC 50 value for active extract. The values were obtained from at least three determinations. Data were analysed using One way ANOVA. *P* < 0.05 was considered to be statistically significant.

## 4. Conclusions

The results of this study indicate that the strong PAF antagonistic activity of *Mitrephora vulpina* was due to phylligenin and quebrachitol which exhibited potent and concentration-dependent inhibitory effects on PAF receptor binding. Toxicity study of the compounds needs to be conducted for their development into therapeutic agents. Phylligenin also showed strong dose-dependent inhibitory activity on platelet aggregation induced by AA and ADP. However, its exact mechanism is unclear although it has potential effect like aspirin, *i.e*. due to the inhibition of thromboxane A_2_ formation. More investigations need to be carried out, including the use of a wider panel of agonists such as PAF and thrombin, in order to elucidate its mechanisms of action.

## Supplementary Materials

Supplementary File 1
