# Correction: Moharam *et al.*, Inhibitory Effects of Phylligenin and Quebrachitol Isolated from *Mitrephora vulpina* on Platelet Activating Factor Receptor Binding and Platelet Aggregation. *Molecules* 2010, *15*, 7840-7848

**DOI:** 10.3390/molecules19033848

**Published:** 2014-03-24

**Authors:** Bushra Abdulkarim Moharam, Ibrahim Jantan, Juriyati Jalil, Khozirah Shaari

**Affiliations:** 1 Faculty of Pharmacy, Universiti Kebangsaan Malaysia, Jalan Raja Muda Abdul Aziz, Kuala Lumpur 50300, Malaysia; 2 Institute of Bioscience, Universiti Putra Malaysia 43400 Serdang, Selangor, Malaysia

The authors wish to inform readers that there are several minor errors and omissions in the chemical structures shown in Figure 1 of this paper [1]. While our phylligenin structure did show the correct 4*S* stereochemistry at the point of attachment of the 3,4-dimethoxyphenyl substituent, the 1*R* stereochemistry of the 2-methoxyphenol attachment point was omitted. In the structure of quebrachitol the stereochemistry that distinguishes this particular compound from other O-methylinositol isomers was not indicated. Finally, a double bond was missing in the structure of oxoputerine. The corrected Figure 1 is shown below.

**Figure 1:**
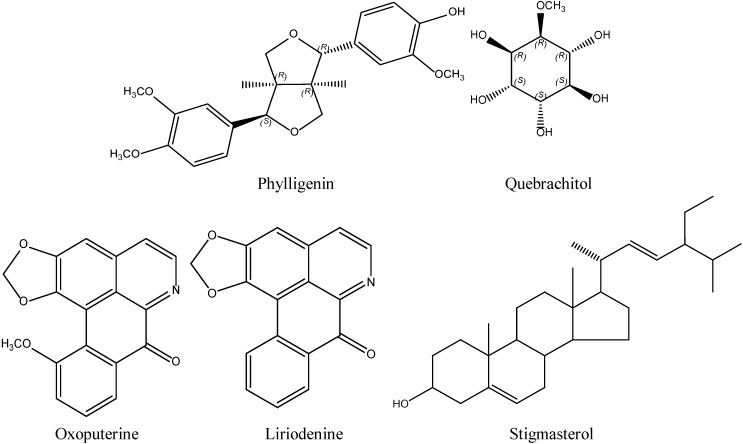
Structures of compounds from *Mitrephora vulpina*.

Finally, we have been alerted by Prof. Christophe Wiart (University of Nottingham) that the taxonomy of several of the *Mitrephora* species mentioned in our paper, including the title species *M. vulpina*, has changed, and the names *M. vulpina* C.E.C. Fisch, *M. zippeliana* and *M. diversifolia* are no longer used, while the species *M. thorelii* Pierre is now *M. tomentosa* Hook f. & Thomson. Readers are advised to consult a recent monograph on this genus [1] for the most current information.
