# Supplementary material for: Inhibitory Effects of Phylligenin and Quebrachitol Isolated from Mitrephora vulpina on Platelet Activating Factor Receptor Binding and Platelet Aggregation
Source: Molecules. 2010 Nov 3;15(11):7840–8. doi: 10.3390/molecules15117840 (PMC6259456; doi:10.3390/molecules15117840)
Supplement: Supplementary File 1 [file molecules-15-07840-s001.pdf]

Molecules **2014**, *19*, 3848-3849; doi:10.3390/molecules19033848

OPEN ACCESS

molecules

ISSN 1420-3049

www.mdpi.com/journal/molecules

Correction

Correction: Moharam *et al.*, Inhibitory Effects of Phylligenin and Quebrachitol Isolated from *Mitrephora vulpina* on Platelet Activating Factor Receptor Binding and Platelet Aggregation. *Molecules* 2010, *15*, 7840-7848

Bushra Abdulkarim Moharam [1], Ibrahim Jantan [1,*], Juriyati Jalil [1] and Khozirah Shaari [2]

[1] Faculty of Pharmacy, Universiti Kebangsaan Malaysia, Jalan Raja Muda Abdul Aziz, Kuala Lumpur 50300, Malaysia

[2] Institute of Bioscience, Universiti Putra Malaysia 43400 Serdang, Selangor, Malaysia

* Author to whom correspondence should be addressed; E-Mail: ibj@pharmacy.ukm.my; Tel.: +603-9289-7315; Fax: +603-9289-3271.

Received: 4 March 2014 / Accepted: 11 March 2014 / Published: 24 March 2014

The authors wish to inform readers that there are several minor errors and omissions in the chemical structures shown in Figure 1 of this paper [1]. While our phylligenin structure did show the correct 4*S* stereochemistry at the point of attachment of the 3,4-dimethoxyphenyl substituent, the 1*R* stereochemistry of the 2-methoxyphenol attachment point was omitted. In the structure of quebrachitol the stereochemistry that distinguishes this particular compound from other O-methylinositol isomers was not indicated. Finally, a double bond was missing in the structure of oxoputerine. The corrected Figure 1 is shown below.

Figure 1. Structures of compounds from *Mitrephora vulpina.*

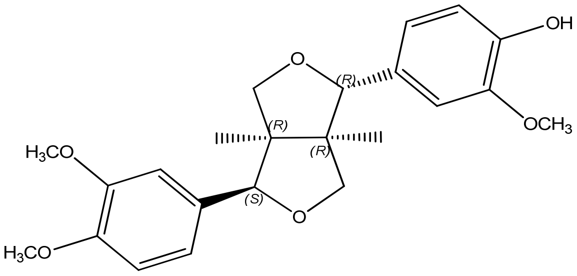

Phylligenin

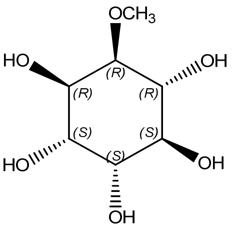

Quebrachitol

Figure 1. *Cont.*

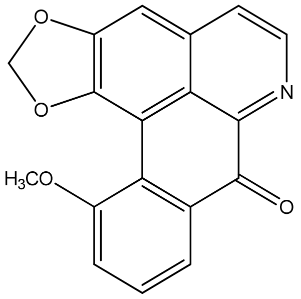

Oxoputerine

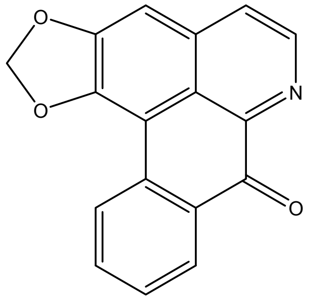

Liriodenine

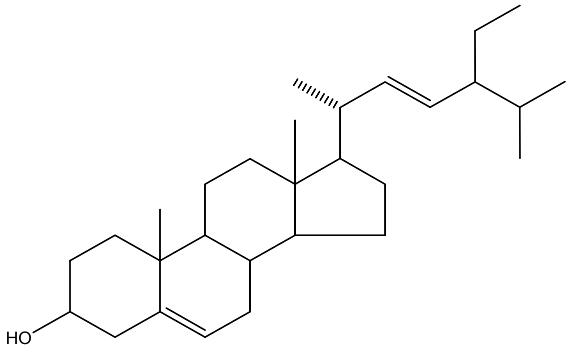

Stigmasterol

Finally, we have been alerted by Prof. Christophe Wiart (University of Nottingham) that the taxonomy of several of the *Mitrephora* species mentioned in our paper, including the title species *M. vulpina*, has changed, and the names *M. vulpina* C.E.C. Fisch, *M. zippeliana* and *M. diversifolia* are no longer used, while the species *M. thorelii* Pierre is now *M. tomentosa* Hook f. & Thomson. Readers are advised to consult a recent monograph on this genus [1] for the most current information.

References

1. Moharam, B.A.; Jantan, I.; Jalil, J.; Shaari, K. Inhibitory Effects of Phylligenin and Quebrachitol Isolated from Mitrephora vulpina on Platelet Activating Factor Receptor Binding and Platelet Aggregation. *Molecules* **2010**, *15*, 7840–7848.
2. Weerasooriya, A.D.; Saunders, R.M.K. *Systematic Botany Monographs: Monograph of Mitrephora (Annonaceae)*; American Society of Plant Taxonomists: Laramie, WY, USA, 2010; Volume 90.

© 2014 by the authors; licensee MDPI, Basel, Switzerland. This article is an open access article distributed under the terms and conditions of the Creative Commons Attribution license (http://creativecommons.org/licenses/by/3.0/).
